# Sub-millisecond time-resolved SAXS using a continuous-flow mixer and X-ray microbeam

**DOI:** 10.1107/S0909049513021833

**Published:** 2013-10-01

**Authors:** Rita Graceffa, R. Paul Nobrega, Raul A. Barrea, Sagar V. Kathuria, Srinivas Chakravarthy, Osman Bilsel, Thomas C. Irving

**Affiliations:** a BioCAT, CSRRI and Department BCS, Illinois Institute of Technology, 3101 South Dearborn, Chicago, IL 60616, USA; b Department of Biochemistry and Molecular Pharmacology, University of Massachusetts Medical School, 364 Plantation Street, LRB 919, Worcester, MA 01605, USA

**Keywords:** micro-SAXS, time-resolved, protein folding

## Abstract

Small-angle X-ray scattering (SAXS) is a well established technique to probe the nanoscale structure and interactions in soft matter. It allows one to study the structure of native particles in near physiological environments and to analyze structural changes in response to variations in external conditions. The combination of microfluidics and SAXS provides a powerful tool to investigate dynamic processes on a molecular level with sub-millisecond time resolution. Reaction kinetics in the sub-millisecond time range has been achieved using continuous-flow mixers manufactured using micromachining techniques. The time resolution of these devices has previously been limited, in part, by the X-ray beam sizes delivered by typical SAXS beamlines. These limitations can be overcome using optics to focus X-rays to the micrometer size range providing that beam divergence and photon flux suitable for performing SAXS experiments can be maintained. Such micro-SAXS in combination with microfluidic devices would be an attractive probe for time-resolved studies. Here, the development of a high-duty-cycle scanning microsecond-time-resolution SAXS capability, built around the Kirkpatrick–Baez mirror-based microbeam system at the Biophysics Collaborative Access Team (BioCAT) beamline 18ID at the Advanced Photon Source, Argonne National Laboratory, is reported. A detailed description of the microbeam small-angle-scattering instrument, the turbulent flow mixer, as well as the data acquisition and control and analysis software is provided. Results are presented where this apparatus was used to study the folding of cytochrome *c*. Future prospects for this technique are discussed.

## Abstract 2

The development of a high-duty-cycle microsecond time-resolution SAXS capability at the Biophysics Collaborative Access Team beamline (BioCAT) 18ID at the Advanced Photon Source, Argonne National Laboratory, USA, is reported.

## 1. Introduction

Time-resolved studies of structural changes in biological macromolecules are of fundamental importance in understanding their biological function. One important application is the study of protein and RNA folding, processes that involve a large dynamic range in both length and time scales, with many important processes involved in hydrophobic collapse occurring on the microsecond to millisecond time scale (Kathuria *et al.*, 2011 ▶; Sosnick & Barrick, 2011 ▶; Svergun & Koch, 2003 ▶; Thirumalai *et al.*, 2001 ▶; Woodson, 2010 ▶). Similarly, ligand and RNA/DNA binding (Wee *et al.*, 2012 ▶), assembly of lipid bilayer structures and nano-particle-based drug delivery systems (Johnson & Prud’homme, 2003 ▶), vesicle formation (Weiss *et al.*, 2005 ▶; Guida, 2010 ▶), protein association (Doyle *et al.*, 2004 ▶) and conformational dynamics (Chattopadhyay *et al.*, 2002 ▶; Werner *et al.*, 2006 ▶; Srajer & Royer, 2008 ▶) also typically occur on a sub-millisecond timescale. The most common way to experimentally investigate such processes is to initiate them by rapid mixing of individual components and/or changing the solvent, pH or ionic strength, using various microfluidic devices. Fluorescence (*e.g.* FRET) and other optical techniques have been typically used to access kinetics on these timescales using such devices (Lipman *et al.*, 2003 ▶; Schuler & Eaton, 2008 ▶; Roder & Shastry, 1999 ▶). Fluorescence measurements, however, report on changes in local structure rather than global shape changes and frequently require exogenous fluorescent labels. Small-angle X-ray scattering (SAXS), in contrast, can probe the macromolecular size, shape and oligomeric state of macromolecular complexes without the need for extrinsic labeling (Svergun & Koch, 2003 ▶) making it a versatile tool for quantitatively probing transiently populated protein structural states.

While stopped-flow techniques with SAXS have been used for quite some time for kinetic studies (Eliezer *et al.*, 1995 ▶; Roh *et al.*, 2010 ▶), time resolution has been limited by the dead-time of current commercial devices to about ∼0.5 ms. Various kinds of continuous-flow mixers, however, have the potential to go much faster (Kathuria *et al.*, 2011 ▶). Among these approaches, turbulent mixing methods (Roder *et al.*, 2004 ▶) have the advantage of being able to take advantage of all of the delivered flux in the X-ray beam for the best signal-to-noise ratio (S/N). Turbulence-based mixers use high-Reynolds-number flow (Re > 10^3^) in a micromachined channel to reduce the size of the largest eddies to ∼0.1 µm (Roder *et al.*, 2004 ▶; Regenfuss *et al.*, 1985 ▶). The rate-limiting step in mixing is diffusion over this distance, which is determined by the diffusion time, *t*_d_ = λ^2^/*D*, where λ is the diffusion length and *D* is the translational diffusion coefficient (typically ∼10^−5^ cm^2^ s^−1^ for small molecules and ∼10^−7^ cm^2^ s^−1^ for biological macromolecules). Small-solvent molecules and additives, such as chemical denaturants and metal ions, diffuse over this length scale within 10 µs. The plug flow in turbulent mixing gives rise to a relatively uniform reaction time in the channel orthogonal to the flow direction, making interfacing to SAXS relatively straightforward (Akiyama *et al.*, 2002 ▶; Arai *et al.*, 2007 ▶). Another advantage of turbulent mixers is that the linear flow velocity is typically ∼4 m s^−1^ (or ∼250 µs mm^−1^), resulting in a small dead-time (∼150–200 µs). Because of the high flow rates, sample can tolerate synchrotron X-ray flux without radiation damage and heating effects, delivering S/N comparable with those from static equilibrium experiments. In previously reported experiments, typical beam dimensions (35–50 µm FWMH in the vertical) limited time resolution by requiring relatively wide channels, ∼300 µm to avoid scattering from the tails of the beam hitting the edges of the channel (Arai *et al.*, 2007 ▶; Wu *et al.*, 2008 ▶). Better time resolution can be achieved using a smaller X-ray beam focal spot that would permit using narrower mixer channels. Another limitation was the low duty cycle (∼10%) dictated by the slow readout of the CCD-based area detectors used in these earlier experiments. Because the sample is continuously flowing, sample consumption is very high, prohibitively so for many experiments. Typically, 50–100 mg has been required for the acquisition of a single time point with ∼15 × 1 s exposures. Together these limitations restricted a given experiment to about 6–12 data points (Wu *et al.*, 2008 ▶) whereas ∼100 points are required to allow separation of co-existing species, very important for folding studies, using singular value decomposition approaches (Henry & Hofrichter, 1992 ▶).

Here, we present a new approach that provides improved time resolution along with better data quality with lower sample consumption by combining an improved continuous-flow turbulent mixer device with a new micro-SAXS instrument built on the BioCAT beamline 18ID at the Advanced Photon Source, Argonne National Laboratory. By scanning the mixer continuously across the beam in synchrony with continuous readout of the X-ray data sample consumption, radiation damage and heating artifacts are reduced and the duty cycle is increased from less than 10% to higher than 80%. The ability to use narrower mixer channels has increased the time resolution from ∼200 µs to 100 µs. Sample consumption is now ∼2–3 mg protein per data point as opposed to the 100 mg per data point required in earlier studies. Future prospects for this technology are discussed.

## 2. Experimental approach

### 2.1. X-ray optics

**Figure 1:**
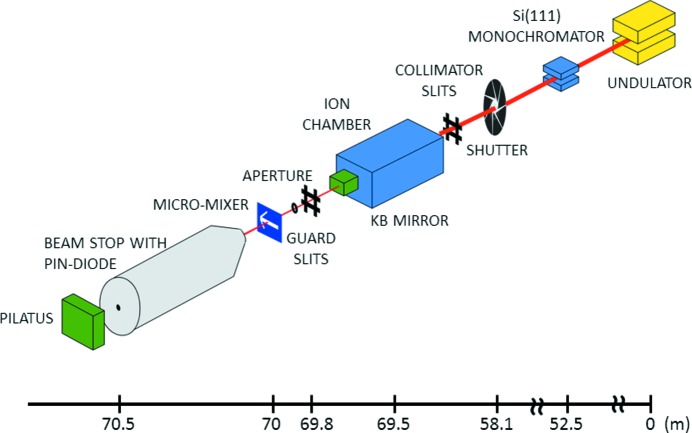
Schematic drawing of the beamline optics and micro-SAXS set-up. The X-ray beam travels from the right to the left in the figure and focuses to 20 µm × 5 µm on the mixer. The microchannel is scanned horizontally in synchrony with continuous readout of scattering patterns using a Pilatus detector.

The main components of the BioCAT undulator beamline 18ID comprise an APS undulator type ‘A’, a cryo-cooled double-crystal Si(111) monochromator, and a Kirkpatrick–Baez mirror bender system (‘KB system’) of the University of Chicago design (Eng *et al.*, 1998 ▶) for production of high-flux microbeams with an adjustable focal distance of 300–500 mm (Barrea *et al.*, 2010 ▶). The KB system, mounted inside a helium-filled chamber to protect the surface of the mirrors, is located at the end of the experimental hutch D of beamline 18ID, 70 m downstream from the undulator source. The size of the incoming beam is defined by a set of slits located immediately upstream of the He mirror chamber. The maximum acceptance of the KB mirrors is ∼0.5 mm × 0.5 mm collecting about one-tenth of the total available beam coming off the beamline monochromator for a delivered flux of ∼10^12^ photons s^−1^. The intensity of the microbeam delivered by the KB system is monitored by a small ion chamber mounted just downstream of the He chamber. A second set of vacuum-compatible slits and a pinhole aperture (50–250 µm diameter) located downstream of the ion chamber are used as guard apertures. The slits and the aperture are independently aligned using two separate sets of motorized linear stages (see Fig. 1 ▶). Additional details of the beamline optics can be found elsewhere (Barrea *et al.*, 2006 ▶, 2010 ▶; Fischetti *et al.*, 2004 ▶). For micro-SAXS, an evacuated flight tube, 0.5 m-long, is located immediately downstream of the sample and upstream of the detector. The beam size at the mixer is ∼20 µm × 5 µm FWHM horizontal and vertical, respectively. The transmitted intensity is collected by a pin diode mounted on the 3 mm beam stop. The KB mirrors, being achromatic devices, allow facile changing of the beam energy so that the beam energy can be readily changed between 8 and 12 keV. The usable *Q*-range is 0.015 to 0.35 Å^−1^ at 8 keV and 0.025 to 0.55 Å^−1^ at 12 keV.

### 2.2. Turbulent flow mixer

**Figure 2:**
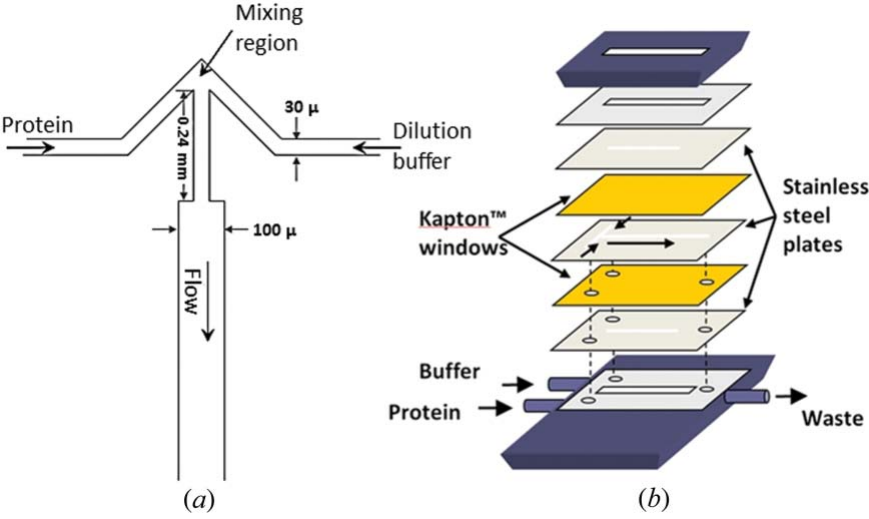
Schematic of the mixing plate and mixer assembly used for continuous-flow mixing. (*a*) An enlarged view of the mixing region is shown. The 100 µm-wide section is the observation region with Kapton windows. Other parts are covered by stainless steel plates precisely machined to match the observation region and seal the mixing region. Channels are 30 µm wide in the mixing region and 100 µm wide in the observation region. (*b*) The assembly of the mixing plate; Kapton windows and matching stainless steel plates are shown. The unit is held together with pressure applied to the 12.5 mm-thick top and bottom stainless steel plates (shown in dark blue). Stainless steel guide pins were used on the base plate to keep the three precisely wire-EDM machined plates in register to within 10 µm during assembly.

A key component of the system is the turbulent mixer (Bilsel *et al.*, 2005 ▶). In order to reduce sample consumption and increase time resolution, a narrow channel is required. A channel width smaller than 100 µm is needed to achieve turbulent mixing with minimum dead-times in the 50 µs time range as demonstrated with fluorescence studies (Bilsel *et al.*, 2005 ▶). This width is also sufficient to avoid scattering from the tails of the intensity distribution of the X-ray microbeam. Scattering intensity will depend on the path length of the X-ray beam through the sample and should be as thick as possible, given the other considerations, in order to maximize S/N. The mixer is constructed, therefore, by wire electric discharge machining channels in a 400 µm-thick stainless steel plate. The mixing region of the plate is a variation of the T-mixer design (Akiyama *et al.*, 2002 ▶) wherein the T-junction is replaced by an arrow-shape (Bilsel *et al.*, 2005 ▶) as shown in Fig. 2 ▶. The channels are 30 µm wide in the mixing region and expand to 100 µm wide in the 20 mm-long observation region. The top and bottom of the channel are formed by 8 µm-thick Kapton films sandwiched by stainless steel plates with an observation window matching the dimensions of the channel in the observation region. With this device, mixing times of 93 µs can be achieved for a 1/10 dilution reaction of 8 *M* urea with water at flow rates of 10–20 ml min^−1^. Since the reaction time after mixing corresponds to the beam position along the 20 mm-long channel, time-resolved SAXS measurements are obtained by scanning the channel across the beam in order to continuously sample time points in synchrony with collection of scattering patterns (see below). At the flow rates currently used (∼120 µs mm^−1^), the beam dimension parallel to the flow direction is negligible in its effect on time resolution. (It would become an issue only if the mixing time was faster than 15 µs.)

### 2.3. Continuous scanning data acquisition

The turbulent flow mixer is mounted on a high-precision stage that allows scanning of the mixer across the X-ray beam in synchrony with continuous readout of scattering patterns using a pixel array detector (Pilatus 100k, Dectris). The reagent solutions flow into the microfluidic device, where they mix to initiate the reaction. Since the reagents are flowing at a known constant flow velocity (±1 µl min^−1^), the distance from the mixing point along the microchannel corresponds to a known time after mixing. The evolution of conformational changes of the products after mixing is probed by translating the microchannel horizontally (along the flow direction) across the X-ray beam and collecting the SAXS patterns. This data acquisition strategy is in contrast to earlier experiments where protein and blank scattering curves were acquired at one position along the channel prior to acquiring another time point (Arai *et al.*, 2007 ▶; Wu *et al.*, 2008 ▶). This process is very inefficient: the sample is continuously flowing to keep the steady-state flow condition (pressure variation of ±1 p.s.i. and flow variation of ±1 µl min^−1^) while the sample stage is moving with the shutter closed and no data are collected. By using continuous scanning and continuous data collection, data are collected much more quickly and sample consumption greatly reduced. The duty cycle (*i.e.* the percentage of time sample flowing that is actually collecting scattering data) is therefore maximized. The fast scanning mode currently requires 2–3 mg of protein per point for each of a total of 90 data points collected in a time series while the previous approach required 50–100 mg per point for a total of 6–10 data points in a time series. Additional advantages of scanning are the avoidance of radiation damage to the sample and heating effects on the micromixer that could compromise the data quality. Scanning is also advantageous because it avoids repetitively hitting the same spot on the Kapton window. This significantly increases the longevity of the mixer. Once assembled, the mixer can be used for multiple experiments without disassembly.

**Figure 3:**
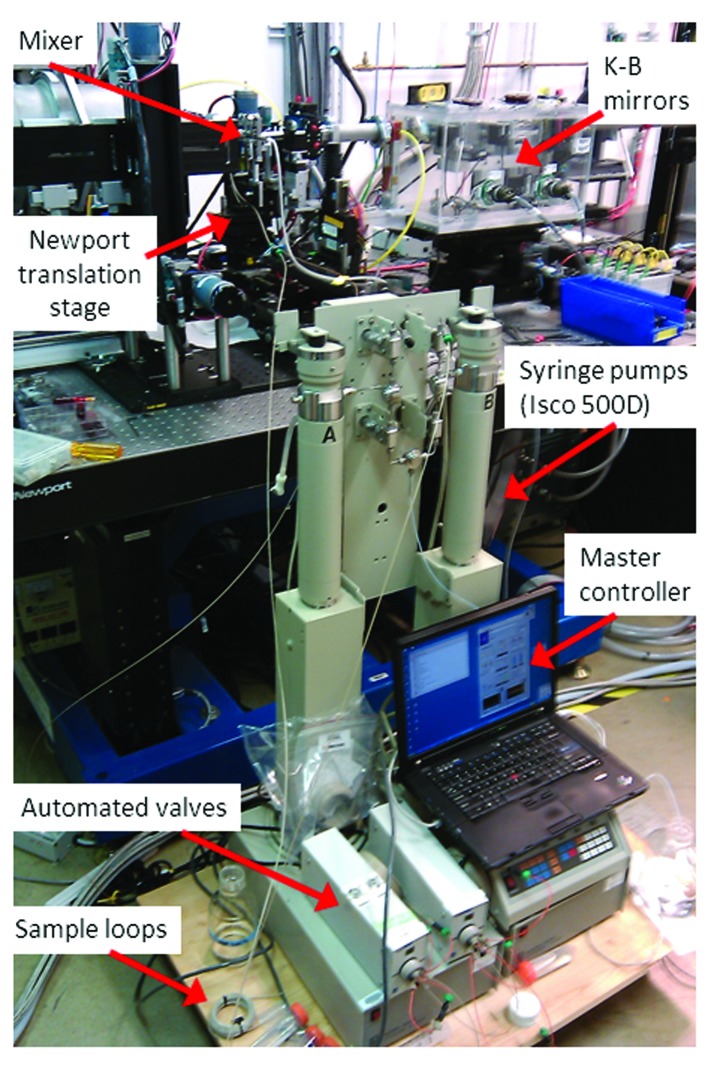
Beamline set-up during an experiment. The Isco 500D pumps and the master control flush the reagents to the micromixer. After the beam is focused by the KB mirror and cleaned by guard slits and aperture and hits the microchannel. The scattering signal is collected by a Pilatus detector, placed on the back of the flight tube.

Mixer alignment and positioning is performed by a set of high-precision (0.1 µm resolution) motorized (Newport ILS50pp) stages mounted in *XY* configuration and a rotary stage (Fig. 3 ▶). The scanning software is based on the EPICs control system used at the APS. Python custom software provides synchronization with the shutter, Pilatus detector, fluid flow control as well as providing motor position time stamps. Ion chamber and transmission monitor readings are recorded synchronously with the scanning and binned to correspond with each image along the scan and used for normalization.

The reagents are delivered to the mixer by two high-pressure pumps (Isco 500D, Teledyne). The data acquisition starts when the flow inside the mixer has stabilized. The shutter opens, allowing the X-rays to hit the microchannel at the mixing point and the Pilatus detector starts to continuously read out (5 frames s^−1^, 190 ms-long frames, 4 ms readout between frames, 6 ms gap). The 20 mm-long microchannel moves with respect to the beam position with a speed of 1 mm s^−1^. When the whole mixer is scanned and the end of the channel furthest from the mixing point reaches just short of the beam position, the shutter closes, the detector stops acquiring and the mixer is moved back to its initial position with a speed of 10 mm s^−1^. During data collection, the mixer is continuously moving while the solutions inside are constantly flowing. Approximately 10–15 scans are collected with blank and with protein to obtain the desired S/N. Each scan takes a total of 21 s, comprising 19 s of data collection plus 2 s wait time per scan. This protocol gives 80 to 100 time points with 200 ms acquisition per point and 20 s per full scan (∼86% duty cycle). A full kinetic profile from 100 µs to 2.4 ms with ∼80–100 points can be obtained in about 30 min including loading of the samples.

### 2.4. Data analysis

Analysis software that allows straightforward exporting and batch processing of the data has also been completed. Every segment of the mixer generates different parasitic scattering due to irregularities or scratches on the Kapton X-ray windows. This needs to be masked out for radial integration of the scattering data. Manually generating such a mask at each point is labor intensive. Using the *FIT2D* program (http://www.esrf.eu/computing/scientific/FIT2D/), a basic mask is drawn and, *via* thresholding of higher values using an automatic Python/*FIT2D* procedure, a specific mask at every point is superimposed. The two-dimensional patterns are integrated, *via* automated macros in *FIT2D*, to obtain the azimuthally averaged intensities and corresponding errors as a function of scattering vector. These are then divided by the transmission recorded for every image. Patterns recorded on the same channel segment (corresponding to the same reaction time) for repeating scans are averaged. The averaged blank scattering curves are subtracted from the protein curves at each point along the channel.

## 3. Results

**Figure 4:**
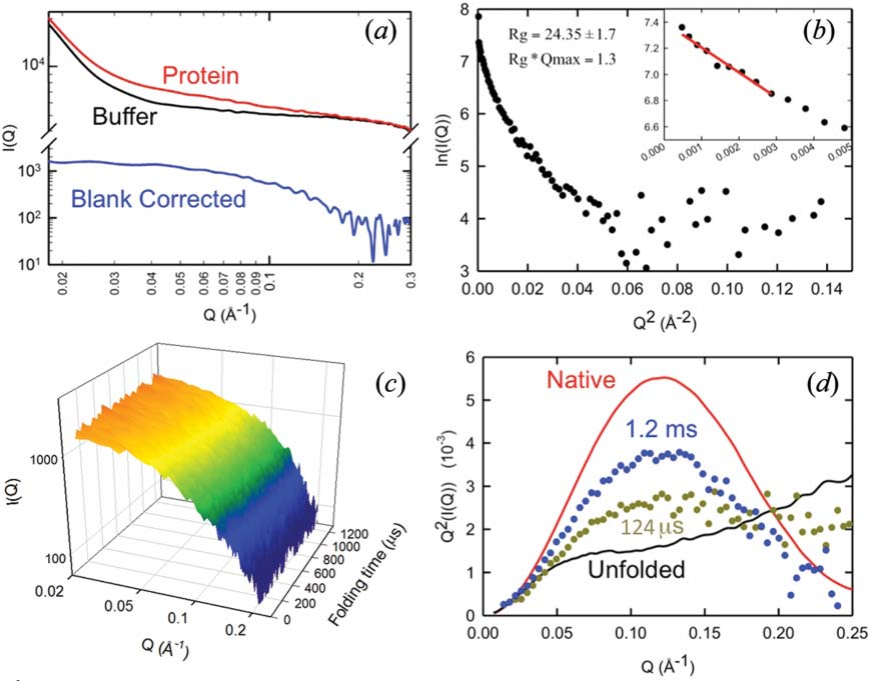
Representative continuous-flow micro-SAXS data collected on horse heart cytochrome *c*. Refolding from the random-coil-like state (4.5 *M* GdnHCl) was initiated by tenfold dilution of GdnHCl with buffer using the continuous-flow mixer. (*a*) Raw data for protein (red) and buffer (black) at a representative time point (100 µs) along the channel. The blank subtracted data are shown in blue. (*b*) Representative Guinier fit of the data (100–148 µs points averaged). The solid red line is a weighted least-squares Guinier fit. (*c*) The blank-corrected scattering curves for 3.5 mg ml^−1^ cytochrome *c* over the 0.1–1.2 ms time range after initiation of folding. Final conditions are 0.45 *M* GdnHCl, 0.2 *M* imidazole and pH 7.0. Each scattering curve is the average of approximately ten frames of 200 ms exposure. (*d*) Kratky plots at representative time points compared with measurements taken under equilibrium conditions for folded (red) and unfolded (black) cytochrome *c*. Data from 100 to 148 µs were averaged for a representative plot of the beginning of the channel (gold), and 2.33–2.40 ms were averaged for a representative plot of the end of the channel (blue). Each plot is normalized to *I*_0_.

The micro-SAXS continuous-flow approach was applied to the folding of cytochrome *c*, a well studied model system known to exhibit kinetics in the sub-millisecond time regime (Chan *et al.*, 1997 ▶; Shastry & Roder, 1998 ▶). For these studies, the protein was denatured using 4.5 *M* [GdnHCl] to populate a conformational state with a radius of gyration consistent with a random coil (Kathuria *et al.*, 2011 ▶). A tenfold dilution of this solution with buffer in the continuous-flow mixer gives rise to conditions favoring the native state. Previous continuous-flow SAXS and fluorescence studies have indicated that the transition from a random-coil-like state to the native state occurs with a sub-100 µs kinetic step followed by a 650 µs step (Chan *et al.*, 1997 ▶; Shastry & Roder, 1998 ▶). The raw scattering profiles at the earliest time for which mixing is complete, 100 µs, are shown in Fig. 4(*a*) ▶. The full data set of blank subtracted scattering curves normalized for transmitted beam intensity is shown in Fig. 4(*b*) ▶.

**Figure 5:**
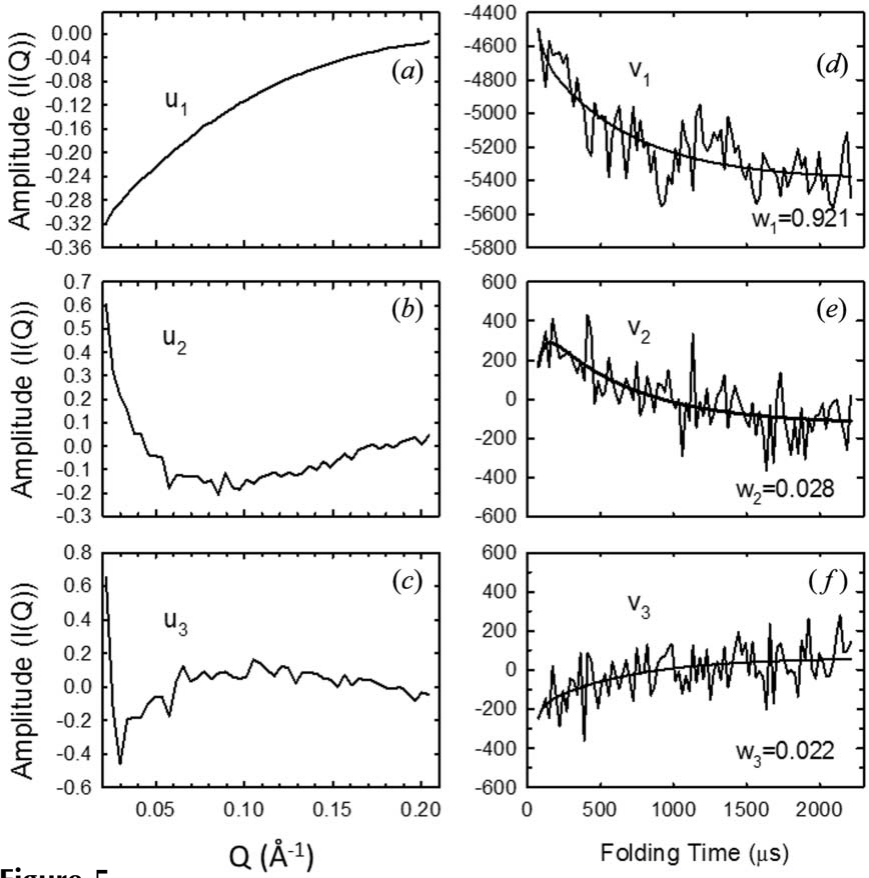
Singular value decomposition of the blank-corrected scattering data for cytochrome *c*. Experimental conditions are the same as in Fig. 4 ▶. Only the first three basis vectors along the scattering angle and the time axis are shown. The remaining vectors consist of random noise as judged by their autocorrelations and singular values. The data are fit to a double exponential function with time constants of 30.2 and 457 µs.

A valuable feature of a data set such as that shown in Fig. 4 ▶ is that a wide range of length scales, determined by the *Q*-range, can be kinetically monitored during the conformational transition. This sensitivity to the distribution of distances is important for determining whether the process proceeds *via* intermediates or occurs in a single kinetic step. Matrix reduction approaches such as singular value decomposition (SVD) can take advantage of the increased data density with the scanning approach in identifying additional components, if present. A SVD analysis of the data from Fig. 4 ▶ is shown in Fig. 5 ▶. Only the first three basis vectors (u1, v1, u2, v2, u3, v3) are necessary to describe the complete data set (minus the noise). The absence of additional components is consistent with the compaction and the transition to the native state being a highly cooperative process. A global non-linear least-squares fit of the folding time dependence (the v-vectors) is equivalent to a full global fit of the entire data set. SVD effectively performs averaging and filtering of the data. The full sampling of the kinetics also allows the user to model the kinetics and extract the true species scattering curves from the data (Akiyama *et al.*, 2002 ▶). Structural modeling of the data with a variety of currently available algorithms (Svergun & Koch, 2003 ▶) is greatly facilitated by having separated out the mixture of species contributing to the scattering curve at any given time point along the channel.

## 4. Conclusions and future prospects

We have demonstrated a continuous-flow micro-SAXS apparatus that is capable of achieving time resolutions of 100 µs with *Q*-ranges appropriate for macromolecules the size of small proteins. This *Q*-range is also suitable for kinetic studies of soft-condensed-matter systems such as micelles and vesicles (Graceffa *et al.*, in preparation). Sample requirements, while still large (∼2 mg per time point, with ∼90 time points total), are achievable for many systems and represent over an order of magnitude improvement in sample utilization. Further optimizations of pump arrangements and reduction of dead volume underway are expected to reduce sample consumption by an additional factor of four to eight so that the total amount of protein will be of the order of 50 mg per experiment or less, bringing it in reach of a much broader range of systems. We are currently evaluating a compound refractive lens optic to replace the KB mirrors. The expected 1 µm × 20 µm (V × H) focal spot will allow use of smaller channels, as is routinely used in fluorescence studies, for higher time resolution (potentially ∼30 µs). The 2 m focal distance (four times longer than with the KB mirrors) will reduce divergence by up to a factor of four, allowing reduction of the minimum *Q* to ∼0.004–0.008 Å^−1^, comparable with the standard BioCAT SAXS instrument. These developments will allow application of these techniques to a very large class of biomedically important problems as well as other applications in the study of soft-condensed matter.
